# Supplementary material for: Perspective on the Use of DNA Repair Inhibitors as a Tool for Imaging and Radionuclide Therapy of Glioblastoma
Source: Cancers (Basel). 2022 Apr 3;14(7):1821. doi: 10.3390/cancers14071821 (PMC8997380; doi:10.3390/cancers14071821)
Supplement: Supplementary file 1 [file cancers-14-01821-s001.zip › cancers-1633092-supplementary.pdf]

Supplementary Material: Perspective on the Use of DNA Repair Inhibitors as a Tool for Imaging and Radionuclide Therapy of Glioblastoma

Liesbeth Everix, Shankari Nair, Cathryn H. S. Driver, Ingeborg Goethals, Mike M. Sathekge,Thomas Ebenhan, Charlot Vandevoorde and Julie Bolcaen

Table S1. Overview of cancer clinical trials of ATMi and ATRi.

Target	Drug	Combined Therapy*	Clinical Phase	Condition/Disease	Biomarker Selection/ Evaluation	Trial Number (Status) Reference
ATM	AZD1390	RT	I	GB, Brain neoplasms, malignant LMD	/	NCT03423628 (r)
ATM	AZD0156	Olaparib/Irinotecan/ Fluorouracil/Leucovorin	I	AST (including glioma)	/	NCT02588105 (anr)
ATM	KU-60019	CK2 (CX4945)	NA	Kidney C	/	NCT03571438 (r)
ATM	M3541	RT	I	ST	/	NCT03225105 (c)
ATR	VX-970 (VE-822, M6620, berzosertib, Merck®)	/	II	AST	ATRX, ATM, HRR, BRCA, MYC, FBXW7, or cyclin E changes; yH2AX, phospho-CHK1	NCT03718091 (anr)
		Carboplatin/Gemcitabine	II	OC, PFTC	/	NCT02595892 (anr)
		Topotecan	II	SCLC and SCC	/	NCT03896503 (s)
		Cisplatin/ Gemcitabine	II	mUC	p53/p21/ERCC2 mut	NCT02567409 (anr)
		Irinotecan	II	GEJC	TP53 mut, DDR mut	NCT03641313 (s)
		Carboplatin/ Docetaxel	II	mCRPC	DNA damage assay, HRR mut	NCT03517969 (anr)
		Carboplatin/Gemcitabine/ Pembrolizumab	I/II	NSCLC	ATM mut	NCT04216316 (r)
		Topotecan	I/II	SCC	/	NCT02487095 (anr)
		Avelumab	I/II	mST/uST	DDR mut	NCT04266912 (r)
		Veliparib/cisplatin	I	AST	yH2AX/RAD51/NBS1/pKAP-1 (DNA damage and apoptosis)	NCT02723864 (c)
		Whole brain RT	I	BM-NSCLC/SCLC/ NET	ATR/CHK1/RAD51	NCT02589522 (anr)
		RT	I	HER2 Negative BC	DNA damage assay, HRR mut	NCT04052555 (r)
		Carboplatin/Gemcitabine	I	OC, PFTC	ATR	NCT02627443 (anr)
		Cisplatin/RT	I	HNSCC	DNA damage	NCT02567422 (anr)
		Irinotecan	I	mST/uST	DDR mut	NCT02595931 (r)
ATR	AZD6738 (ceralasertib, AstraZeneca®)	Cisplatin/Carboplatin/ Etoposide/ Durvalumab	II	Advanced SCLC	/	NCT04699838 (r)
		Durvalumab/Olaparib	II	Biliary Tract C	/	NCT04298021 (r)
		Cediranib/ Olaparib	II	BC	HER2, BRCA	NCT04090567 (r)

		Olaparib/AZD1775/ AZD5363	II	AST	IDH1-2/ATM/CHK2/ APOBEC /MRE11/PTEN/PIK3CA/AKT/ ARID1A/TP53/KRAS	NCT02576444 (anr)
		Durvalumab	II	Biliary Tract C	/	NCT04298008 (r)
		Durvalumab	II	mST	/	NCT03780608 (anr)
		Durvalumab	II	NSCLC	/	NCT03334617 (r)
		Durvalumab	II	Advanced NSCLC	Included (ns)	NCT03833440 (r)
		Durvalumab	II	NSCLC	Included (ns)	NCT02664935 (anr)
		Olaparib	II	Relapsed SCLC	/	NCT03428607(c)
		Olaparib	II	Recurrent OC	BRCA/other HRD mut	NCT03462342 (r)
		Olaparib	II	Gynaecological C	ARID1A loss	NCT04065269 (r)
		Olaparib	II	ST	IDH1/IDH2 mut	NCT03878095 (r)
		Olaparib	II	mCRPC	BRCA, ATM	NCT03787680 (anr)
		Olaparib	II	Advanced SCLC	/	NCT02937818 (anr)
		Olaparib	II	Osteosarcoma	/	NCT04417062 (r)
		Olaparib	II	Advanced BC	ctDNA screening	NCT03182634 (r)
		Olaparib	II	mTNBC	BRCA1-2/HRR mut	NCT03330847 (anr)
		Olaparib	II	Selected ST	BAF250a/ARID1A/ATM	NCT03682289 (r)
		/	II	TNBC	Gene signature	NCT03740893 (r)
		/	II	AST	ATM mut	NCT04564027 (r)
		Paclitaxel	I (+)	Refractory C	/	[1]
		Carboplatin	I (+)	AST	/	[2]
		Olaparib	I	HNSCC	25-gene signature	NCT03022409 (c)
		Gemcitabine	I	AST	/	NCT03669601 (r)
		RT	I	ST	DDR mut	NCT02223923 (unknown)
		DS-8201a	I	AST	HER2/TP53/ATM/RAS	NCT04704661 (r)
		Acalabrutinib	I	CLL	/	NCT03328273 (anr)
ATR	VX-803 (M4344, Merck®)	Carboplatin	I	AST	ARID1A/ATRX/DAXX/ ATM mut	NCT02278250 (c)
		Niraparib	I	PARP-resistant recurrent OC	/	NCT04149145 (anr)
ATR	BAY1895344 (elimusertib, Bayer®)	Pembrolizumab/RT	I	Recurrent HNSCC	/	NCT04576091 (r)
		Pembrolizumab	I	AST	/	NCT04095273 (r)
		/	I	AST and Lymphomas	/	NCT03188965 (r)
		Niraparib	I	AST and OC	/	NCT04267939 (r)
		Fluorouracil/Irinotecan/ Leucovorin	I	Stomach and Intestines C	/	NCT04535401 (r)
		Cisplatin/Gemcitabine	I	AST – UC	/	NCT04491942 (s)
		Gemcitabine	I	Pancreatic C, OC, AST	/	NCT04616534 (s)
		Irinotecan/Topotecan	I	NSCLC, PD-NEC, PDA	/	NCT04514497 (r)
ATR	M1774	Niraparib	I	mST or locally AST/uST	ARIDIA/ATRX/DAXX/ATM mut	NCT04170153 (r)
ATR	RP-3500	Talazoparib/Gemcitabine	I/II	AST	ATRi sensitizing mut, DNA damage	NCT04497116 (r)

Applications in glioblastoma are highlighted in bold. * AZD1775 (WEE1i), AZD5363 (AKTi), Carboplatin/Cisplatin/Docetaxel/Etoposide/Fluorouracil/Gemcitabine/Irinotecan/Paclitaxel (chemotherapy), Leucovorin (folate analog), CX4945 (CK2 inhibitor), DS-8201a/Topotecan (topoisomerase I inhibitor), Cediranib (VEGFRi), Durvalumab/Pembrolizumab/Avelumab

(anti-PD-L1), Olaparib/Talazoparib/Niraparib/Veliparib (PARPi), Acalabrutinib (Bruton's TKI). Abbreviations: anr (active non recruiting), AST (advanced solid tumors), BC (breast cancer), BM (brain metastases), C (cancer), c (completed), CLL (chronic lymphocytic leukemia), CRPC (castration-resistant prostate cancer), DDR (DNA damage repair), GB (glioblastoma), GEJC (gastric or gastroesophageal junction cancer), HNSCC (head and neck squamous cell carcinoma), HRR (homologous recombination repair), LMD (malignant leptomeningeal disease), m (metastatic), mut (mutant/mutated/mutations), NA (not applicable), NET (neuroendocrine tumors), ns (not specified), NSCLC (non-small cell lung cancer), OC (ovarian cancer), PDA (pancreatic adenocarcinoma), PD-NEC (poorly differentiated neuroendocrine carcinoma), PFTC (peritoneal, or fallopian tube cancer), r (recruiting), RT (radiotherapy), s (suspended), SCC (small cell cancer), SCLC (small cell lung cancer), ST (solid tumours), TNBC (triple-negative breast cancer), u (unresectable), UC (urothelial cancer), * "BRCAness" signature.

Table S2. Cancer radiopharmaceuticals targeting DDR kinases.

Target	Drug	Type	Label	Stage	Study Population/Cancer Type	Reference
ATM	AZD1390	SM	[^{11}C]	C	Healthy subjects	[3,4]
	AZD1390 and AZD0156	SM	[^{11}C]	PC	NHP	[5]
ATR	VE-821	SM	[^{18}F]	PC	GB	[6]
CHK1/2	Prexasertib (LY2606368)	SM	[^{14}C]	C	AST	[7]
	LY2603618 (rabusertib)	SM	[^{14}C]	C	AST	[8]
CHK1	GDC-0425	SM	[^{14}C]	PC	Healthy rats	[9]
PARP	Olaparib	SM	[^{18}F]	C	Non-specified brain tumour type	[4]
				C	Non-specified brain tumour type, GB	[10]
				PC	GB	[11–14]
				C	H&N	[4,15]
				PC	PaC, NHP	[16,17]
				PC	OC, BC, PaC, SCLC, H&N, DLBCL	[18–23]
			[^{123}I]	PC	GB	[24]
			[^{131}I]	PC	GB	[25]
			[^{64}Cu]	PC	Mesothelioma	[26]
			[^{11}C]	Syn	/	[27]
	Talazoparib	SM	[^{18}F]	PC	PrC	[28]
	Rucaparib	SM	[^{18}F]	C	OC, PeC, PrC, PaC, BC, FTC	[4,29,30]
				C	GB	[4]
				PC	BC, OC, PrC	[29,31–34]
				PC	PrC	[31]
			[^{125}I]	PC	OC, HCC, BC, NB	[35–38]
			[^{125}I]	PC	BC	[36]
			[^{14}C]	C	AST	[39]
			[^{211}At]	PC	NB	[40,41]
	Pamiparib	SM	[^{14}C]	C	Advanced cancer	[42]
	MAPi*	SM	*[^{123}I]*	PC	GB	[43]
	I2-PARPi	SM	*[^{131}I]*, [^{124}I]	PC	GB	[44]

	Pirenzepine and metabolite LS 75	SM	[^{18}F]	PC	Syn	[45]
DNA-PK	Samotolisib derivative (LY3023414)	SM	[^{14}C]	C	Healthy subjects	[4]
	chromen-4 derivatives	SM	[^{11}C]	Syn	/	[46]

Therapeutic radionuclides are highlighted in italics. Applications in glioblastoma are highlighted in bold. *Meitner-Auger PARP1 inhibitor. Abbreviations: AST (advanced solid tumours), BC (breast cancer), C (clinical study), DLBCL (diffuse large B-cell lymphoma), FTC (fallopian tube cancer), GB (glioblastoma), H&N (head and neck cancer), HCC (hepatocellular carcinoma), NB (neuroblastoma), NHP (non-human primates), OC (ovarian cancer), PaC (pancreas cancer), PC (preclinical study), PeC (peritoneal cancer), PrC (prostate cancer), SCLC (small cell lung cancer), Syn (only synthesized).

References

1. Kim, S.T.; Smith, S.A.; Mortimer, P.; Loembé, A.B.; Cho, H.; Kim, K.M.; Smith, C.; Willis, S.; Irurzun-Arana, I.; Berges, A.; et al. Phase I Study of Ceralasertib (AZD6738), a Novel DNA Damage Repair Agent, in Combination with Weekly Paclitaxel in Refractory Cancer. *Clin Cancer Res* **2021**, *27*, 4700-4709, doi:10.1158/1078-0432.ccr-21-0251.
2. Yap, T.A.; Krebs, M.G.; Postel-Vinay, S.; El-Khouiery, A.; Soria, J.C.; Lopez, J.; Berges, A.; Cheung, S.Y.A.; Irurzun-Arana, I.; Goldwin, A.; et al. Ceralasertib (AZD6738), an oral ATR kinase inhibitor, in combination with carboplatin in patients with advanced solid tumors: a Phase I study. *Clin Cancer Res* **2021**, *27*, 5213-5224, doi:10.1158/1078-0432.ccr-21-1032.
3. Jucaite, A.; Stenkrona, P.; Cselényi, Z.; De Vita, S.; Buil-Bruna, N.; Varnäs, K.; Savage, A.; Varrone, A.; Johnström, P.; Schou, M.; et al. Brain exposure of the ATM inhibitor AZD1390 in humans-a positron emission tomography study. *Neuro Oncol* **2021**, *23*, 687-696, doi:10.1093/neuonc/noaa238.
4. Clinicaltrials.gov. Available online: https://clinicaltrials.gov/ct2/home (accessed on 7 October 2021).
5. Durant, S.T.; Zheng, L.; Wang, Y.; Chen, K.; Zhang, L.; Zhang, T.; Yang, Z.; Riches, L.; Trinidad, A.G.; Fok, J.H.L.; et al. The brain-penetrant clinical ATM inhibitor AZD1390 radiosensitizes and improves survival of preclinical brain tumor models. *Sci Adv* **2018**, *4*, eaat1719, doi:10.1126/sciadv.aat1719.
6. Carlucci, G.; Carney, B.; Sadique, A.; Vansteene, A.; Tang, J.; Reiner, T. Evaluation of [(18)F]-ATRi as PET tracer for in vivo imaging of ATR in mouse models of brain cancer. *Nucl Med Biol* **2017**, *48*, 9-15, doi:10.1016/j.nucmedbio.2017.01.002.
7. Wickremsinhe, E.R.; Hynes, S.M.; Payne, C.D.; Guo, Y.; Cassidy, K.C. Disposition of [(14)C]LY2606368 following intravenous administration in patients with advanced and/or metastatic solid tumours. *Xenobiotica* **2020**, *50*, 793-804, doi:10.1080/00498254.2019.1702736.
8. Wickremsinhe, E.R.; Hynes, S.M.; Palmieri, M.D.; Mitchell, M.I.; Abraham, T.L.; Rehmel, J.F.; Chana, E.; Jost, L.M.; Cassidy, K.C. Disposition and metabolism of LY2603618, a Chk-1 inhibitor following intravenous administration in patients with advanced and/or metastatic solid tumors. *Xenobiotica* **2014**, *44*, 827-841, doi:10.3109/00498254.2014.900589.
9. Takahashi, R.H.; Halladay, J.S.; Siu, M.; Chen, Y.; Hop, C.E.; Khojasteh, S.C.; Ma, S. Novel Mechanism of Decyanation of GDC-0425 by Cytochrome P450. *Drug Metab Dispos* **2017**, *45*, 430-440, doi:10.1124/dmd.116.074336.
10. Young, R.J.; Demétrio De Souza França, P.; Pirovano, G.; Piotrowski, A.F.; Nicklin, P.J.; Riedl, C.C.; Schwartz, J.; Bale, T.A.; Donabedian, P.L.; Kossatz, S.; et al. Preclinical and first-in-human-brain-cancer applications of [(18)F]poly (ADP-ribose) polymerase inhibitor PET/MR. *Neurooncol Adv* **2020**, *2*, vdaa119, doi:10.1093/noajnl/vdaa119.
11. Donabedian, P.L.; Kossatz, S.; Engelbach, J.A.; Jannetti, S.A.; Carney, B.; Young, R.J.; Weber, W.A.; Garbow, J.R.; Reiner, T. Discriminating radiation injury from recurrent tumor with [(18)F]PARPi and amino acid PET in mouse models. *EJNMMI Res* **2018**, *8*, 59, doi:10.1186/s13550-018-0399-z.
12. Carlucci, G.; Carney, B.; Brand, C.; Kossatz, S.; Irwin, C.P.; Carlin, S.D.; Keliher, E.J.; Weber, W.; Reiner, T. Dual-Modality Optical/PET Imaging of PARP1 in Glioblastoma. *Mol Imaging Biol* **2015**, *17*, 848-855, doi:10.1007/s11307-015-0858-0.
13. Carney, B.; Carlucci, G.; Salinas, B.; Di Gialleonardo, V.; Kossatz, S.; Vansteene, A.; Longo, V.A.; Bolaender, A.; Chiosis, G.; Keshari, K.R.; et al. Non-invasive PET Imaging of PARP1 Expression in Glioblastoma Models. *Mol Imaging Biol* **2016**, *18*, 386-392, doi:10.1007/s11307-015-0904-y.
14. Zmuda, F.; Blair, A.; Liuzzi, M.C.; Malviya, G.; Chalmers, A.J.; Lewis, D.; Sutherland, A.; Pimlott, S.L. An (18)F-Labeled Poly(ADP-ribose) Polymerase Positron Emission Tomography Imaging Agent. *J Med Chem* **2018**, *61*, 4103-4114, doi:10.1021/acs.jmedchem.8b00138.
15. Schöder, H.; França, P.D.S.; Nakajima, R.; Burnazi, E.; Roberts, S.; Brand, C.; Grkovski, M.; Mauguen, A.; Dunphy, M.P.; Ghossein, R.A.; et al. Safety and Feasibility of PARP1/2 Imaging with (18)F-PARPi in Patients with Head and Neck Cancer. *Clin Cancer Res* **2020**, *26*, 3110-3116, doi:10.1158/1078-0432.ccr-19-3484.

16. Guibbal, F.; Hopkins, S.L.; Pacelli, A.; Isenegger, P.G.; Mosley, M.; Torres, J.B.; Dias, G.M.; Mahaut, D.; Hueting, R.; Gouverneur, V.; et al. [(18)F]AZD2461, an Insight on Difference in PARP Binding Profiles for DNA Damage Response PET Imaging. *Mol Imaging Biol* **2020**, *22*, 1226-1234, doi:10.1007/s11307-020-01497-6.
17. Reilly, S.W.; Puentes, L.N.; Schmitz, A.; Hsieh, C.J.; Weng, C.C.; Hou, C.; Li, S.; Kuo, Y.M.; Padakanti, P.; Lee, H.; et al. Synthesis and evaluation of an AZD2461 [(18)F]PET probe in non-human primates reveals the PARP-1 inhibitor to be non-blood-brain barrier penetrant. *Bioorg Chem* **2019**, *83*, 242-249, doi:10.1016/j.bioorg.2018.10.015.
18. Reiner, T.; Keliher, E.J.; Earley, S.; Marinelli, B.; Weissleder, R. Synthesis and in vivo imaging of a 18F-labeled PARP1 inhibitor using a chemically orthogonal scavenger-assisted high-performance method. *Angew Chem Int Ed Engl* **2011**, *50*, 1922-1925, doi:10.1002/anie.201006579.
19. Laird, J.; Lok, B.H.; Carney, B.; Kossatz, S.; de Stanchina, E.; Reiner, T.; Poirier, J.T.; Rudin, C.M. Positron-Emission Tomographic Imaging of a Fluorine 18-Radiolabeled Poly(ADP-Ribose) Polymerase 1 Inhibitor Monitors the Therapeutic Efficacy of Talazoparib in SCLC Patient-Derived Xenografts. *J Thorac Oncol* **2019**, *14*, 1743-1752, doi:10.1016/j.jtho.2019.05.032.
20. Carney, B.; Kossatz, S.; Lok, B.H.; Schneeberger, V.; Gangangari, K.K.; Pillarsetty, N.V.K.; Weber, W.A.; Rudin, C.M.; Poirier, J.T.; Reiner, T. Target engagement imaging of PARP inhibitors in small-cell lung cancer. *Nat Commun* **2018**, *9*, 176, doi:10.1038/s41467-017-02096-w.
21. Demétrio de Souza França, P.; Roberts, S.; Kossatz, S.; Guru, N.; Mason, C.; Zanoni, D.K.; Abrahão, M.; Schöder, H.; Ganly, I.; Patel, S.G.; et al. Fluorine-18 labeled poly (ADP-ribose) polymerase1 inhibitor as a potential alternative to 2-deoxy-2-[(18)F]fluoro-d-glucose positron emission tomography in oral cancer imaging. *Nucl Med Biol* **2020**, *84-85*, 80-87, doi:10.1016/j.nucmedbio.2020.01.004.
22. Tang, J.; Salloum, D.; Carney, B.; Brand, C.; Kossatz, S.; Sadique, A.; Lewis, J.S.; Weber, W.A.; Wendel, H.G.; Reiner, T. Targeted PET imaging strategy to differentiate malignant from inflamed lymph nodes in diffuse large B-cell lymphoma. *Proc Natl Acad Sci U S A* **2017**, *114*, E7441-e7449, doi:10.1073/pnas.1705013114.
23. Wilson, T.C.; Xavier, M.A.; Knight, J.; Verhoog, S.; Torres, J.B.; Mosley, M.; Hopkins, S.L.; Wallington, S.; Allen, P.D.; Kersemans, V.; et al. PET Imaging of PARP Expression Using (18)F-Olaparib. *J Nucl Med* **2019**, *60*, 504-510, doi:10.2967/jnumed.118.213223.
24. Zmuda, F.; Malviya, G.; Blair, A.; Boyd, M.; Chalmers, A.J.; Sutherland, A.; Pimlott, S.L. Synthesis and Evaluation of a Radioiodinated Tracer with Specificity for Poly(ADP-ribose) Polymerase-1 (PARP-1) in Vivo. *J Med Chem* **2015**, *58*, 8683-8693, doi:10.1021/acs.jmedchem.5b01324.
25. Jannetti, S.A.; Carlucci, G.; Carney, B.; Kossatz, S.; Shenker, L.; Carter, L.M.; Salinas, B.; Brand, C.; Sadique, A.; Donabedian, P.L.; et al. PARP-1-Targeted Radiotherapy in Mouse Models of Glioblastoma. *J Nucl Med* **2018**, *59*, 1225-1233, doi:10.2967/jnumed.117.205054.
26. Huang, T.; Hu, P.; Banizs, A.B.; He, J. Initial evaluation of Cu-64 labeled PARPi-DOTA PET imaging in mice with mesothelioma. *Bioorg Med Chem Lett* **2017**, *27*, 3472-3476, doi:10.1016/j.bmcl.2017.05.077.
27. Andersen, T.L.; Friis, S.D.; Audrain, H.; Nordeman, P.; Antoni, G.; Skrydstrup, T. Efficient 11C-carbonylation of isolated aryl palladium complexes for PET: application to challenging radiopharmaceutical synthesis. *J Am Chem Soc* **2015**, *137*, 1548-1555, doi:10.1021/ja511441u.
28. Zhou, D.; Chen, H.; Mpoy, C.; Afrin, S.; Rogers, B.E.; Garbow, J.R.; Katzenellenbogen, J.A.; Xu, J. Radiosynthesis and Evaluation of Talazoparib and Its Derivatives as PARP-1-Targeting Agents. *Biomedicines* **2021**, *9*, 565, doi:10.3390/biomedicines9050565.
29. Makvandi, M.; Pantel, A.; Schwartz, L.; Schubert, E.; Xu, K.; Hsieh, C.J.; Hou, C.; Kim, H.; Weng, C.C.; Winters, H.; et al. A PET imaging agent for evaluating PARP-1 expression in ovarian cancer. *J Clin Invest* **2018**, *128*, 2116-2126, doi:10.1172/jci97992.
30. Michel, L.S.; Dyroff, S.; Brooks, F.J.; Spayd, K.J.; Lim, S.; Engle, J.T.; Phillips, S.; Tan, B.; Wang-Gillam, A.; Bognar, C.; et al. PET of Poly (ADP-Ribose) Polymerase Activity in Cancer: Preclinical Assessment and First In-Human Studies. *Radiology* **2017**, *282*, 453-463, doi:10.1148/radiol.2016161929.
31. Zhou, D.; Xu, J.; Mpoy, C.; Chu, W.; Kim, S.H.; Li, H.; Rogers, B.E.; Katzenellenbogen, J.A. Preliminary evaluation of a novel (18)F-labeled PARP-1 ligand for PET imaging of PARP-1 expression in prostate cancer. *Nucl Med Biol* **2018**, *66*, 26-31, doi:10.1016/j.nucmedbio.2018.08.003.
32. Zhou, D.; Chu, W.; Xu, J.; Jones, L.A.; Peng, X.; Li, S.; Chen, D.L.; Mach, R.H. Synthesis, [^{18}F] radiolabeling, and evaluation of poly (ADP-ribose) polymerase-1 (PARP-1) inhibitors for in vivo imaging of PARP-1 using positron emission tomography. *Bioorg Med Chem* **2014**, *22*, 1700-1707, doi:10.1016/j.bmc.2014.01.019.
33. Sander Effron, S.; Makvandi, M.; Lin, L.; Xu, K.; Li, S.; Lee, H.; Hou, C.; Pryma, D.A.; Koch, C.; Mach, R.H. PARP-1 Expression Quantified by [(18)F]FluorThanatrace: A Biomarker of Response to PARP Inhibition Adjuvant to Radiation Therapy. *Cancer Biother Radiopharm* **2017**, *32*, 9-15, doi:10.1089/cbr.2016.2133.
34. Edmonds, C.E.; Makvandi, M.; Lieberman, B.P.; Xu, K.; Zeng, C.; Li, S.; Hou, C.; Lee, H.; Greenberg, R.A.; Mankoff, D.A.; et al. [(18)F]FluorThanatrace uptake as a marker of PARP1 expression and activity in breast cancer. *Am J Nucl Med Mol Imaging* **2016**, *6*, 94-101.

35. Makvandi, M.; Xu, K.; Lieberman, B.P.; Anderson, R.C.; Effron, S.S.; Winters, H.D.; Zeng, C.; McDonald, E.S.; Pryma, D.A.; Greenberg, R.A.; et al. A Radiotracer Strategy to Quantify PARP-1 Expression In Vivo Provides a Biomarker That Can Enable Patient Selection for PARP Inhibitor Therapy. *Cancer Res* **2016**, *76*, 4516-4524, doi:10.1158/0008-5472.can-16-0416.
36. Anderson, R.C.; Makvandi, M.; Xu, K.; Lieberman, B.P.; Zeng, C.; Pryma, D.A.; Mach, R.H. Iodinated benzimidazole PARP radiotracer for evaluating PARP1/2 expression in vitro and in vivo. *Nucl Med Biol* **2016**, *43*, 752-758, doi:10.1016/j.nucmedbio.2016.08.007.
37. Lee, H.; Riad, A.; Martorano, P.; Mansfield, A.; Samanta, M.; Batra, V.; Mach, R.H.; Maris, J.M.; Pryma, D.A.; Makvandi, M. PARP-1-Targeted Auger Emitters Display High-LET Cytotoxic Properties In Vitro but Show Limited Therapeutic Utility in Solid Tumor Models of Human Neuroblastoma. *J Nucl Med* **2020**, *61*, 850-856, doi:10.2967/jnumed.119.233965.
38. Riad, A.; Gitto, S.B.; Lee, H.; Winters, H.D.; Martorano, P.M.; Hsieh, C.J.; Xu, K.; Omran, D.K.; Powell, D.J., Jr.; Mach, R.H.; et al. PARP Theranostic Auger Emitters Are Cytotoxic in BRCA Mutant Ovarian Cancer and Viable Tumors from Ovarian Cancer Patients Enable Ex-Vivo Screening of Tumor Response. *Molecules* **2020**, *25*, 6029, doi:10.3390/molecules25246029.
39. Liao, M.; Watkins, S.; Nash, E.; Isaacson, J.; Etter, J.; Beltman, J.; Fan, R.; Shen, L.; Mutlib, A.; Kemeny, V.; et al. Evaluation of absorption, distribution, metabolism, and excretion of [(14)C]-rucaparib, a poly(ADP-ribose) polymerase inhibitor, in patients with advanced solid tumors. *Invest New Drugs* **2020**, *38*, 765-775, doi:10.1007/s10637-019-00815-2.
40. Makvandi, M.; Lee, H.; Puentes, L.N.; Reilly, S.W.; Rathi, K.S.; Weng, C.C.; Chan, H.S.; Hou, C.; Raman, P.; Martinez, D.; et al. Targeting PARP-1 with Alpha-Particles Is Potently Cytotoxic to Human Neuroblastoma in Preclinical Models. *Mol Cancer Ther* **2019**, *18*, 1195-1204, doi:10.1158/1535-7163.mct-18-0837.
41. Reilly, S.W.; Makvandi, M.; Xu, K.; Mach, R.H. Rapid Cu-Catalyzed [(211)At]Astatination and [(125)I]Iodination of Boronic Esters at Room Temperature. *Org Lett* **2018**, *20*, 1752-1755, doi:10.1021/acs.orglett.8b00232.
42. Mu, S.; Palmer, D.; Fitzgerald, R.; Andreu-Vieyra, C.; Zhang, H.; Tang, Z.; Su, D.; Sahasranaman, S. Human Mass Balance and Metabolite Profiling of [(14) C]-Pamiparib, a Poly (ADP-Ribose) Polymerase Inhibitor, in Patients With Advanced Cancer. *Clin Pharmacol Drug Dev* **2021**, *10*, 1108 - 1120, doi:10.1002/cpdd.943.
43. Pirovano, G.; Jannetti, S.A.; Carter, L.M.; Sadique, A.; Kossatz, S.; Guru, N.; Demétrio De Souza França, P.; Maeda, M.; Zeglis, B.M.; Lewis, J.S.; et al. Targeted Brain Tumor Radiotherapy Using an Auger Emitter. *Clin Cancer Res* **2020**, *26*, 2871-2881, doi:10.1158/1078-0432.ccr-19-2440.
44. Salinas, B.; Irwin, C.P.; Kossatz, S.; Bolaender, A.; Chiosis, G.; Pillarsetty, N.; Weber, W.A.; Reiner, T. Radioiodinated PARP1 tracers for glioblastoma imaging. *EJNMMI Res* **2015**, *5*, 123, doi:10.1186/s13550-015-0123-1.
45. Riss, P.J., Soskic, V., Schrattenholz, A., and Roesch, F. Synthesis and radiosynthesis of N5-[18F]fluoroethyl-pirenzepine and its metabolite N5-[18F]fluoroethyl-LS 75. *J Labelled Comp Radiopharm* **2009**, *52*, 576–579, doi:10.1002/jlcr.1687.
46. Gao, M.; Wang, M.; Miller, K.D.; Zheng, Q.H. Simple synthesis of carbon-11-labeled chromen-4-one derivatives as new potential PET agents for imaging of DNA-dependent protein kinase (DNA-PK) in cancer. *Appl Radiat Isot* **2012**, *70*, 1558-1563, doi:10.1016/j.apradiso.2012.04.023.

Publisher's Note: MDPI stays neutral with regard to jurisdictional claims in published maps and institutional affiliations.

© 2022 by the authors. Licensee MDPI, Basel, Switzerland. This article is an open access article distributed under the terms and conditions of the Creative Commons Attribution (CC BY) license (http://creativecommons.org/licenses/by/4.0/).
